# Survival of eight LLIN brands 6, 12, 24 and 36 months after a mass distribution campaign in rural and urban settings in Senegal

**DOI:** 10.1186/s12889-022-13051-w

**Published:** 2022-04-11

**Authors:** Mbaye Diouf, Babacar Thiendella Faye, El Hadji Diouf, Abdoulaye Kane Dia, Abdoulaye Konate, Fatou Ba Fall, Doudou Sene, Mame Birame Diouf, Libasse Gadiaga, Lassana Konate, Demba Anta Dione, Roger Clément Tine, Ousmane Faye

**Affiliations:** 1 Laboratory of Vector and Parasite Ecology (UCAD), Dakar, Senegal; 2 Laboratory of Parasitology (UCAD), Dakar, Senegal; 3 National Malaria Control Program (NMCP/Senegal), Dakar, Senegal; 4 President’s Malaria Initiative/ United State Agency International Development/Senegal (USAID/PMI), Dakar, Senegal; 5 Health and Development Solution-Africa (HDS-Africa/Dakar), Dakar, Senegal

**Keywords:** LLIN, Survival, Retention, Median survival time, Senegal

## Abstract

**Background:**

Long lasting insecticidal nets (LLIN) are one of the core components of global malaria prevention and control. The lifespan of LLIN varies widely depending on the population or environment, and randomized studies are required to compare LLIN inaccording to arbitrary thresholds

households under different field conditions. This study investigated survival of different LLIN brands in Senegal.

**Methods:**

Ten thousand six hundred eight LLINs were distributed in five regions, each stratified by rural and urban setting. As part of the longitudinal follow-up, 2222 nets were randomly sampled and monitored from 6 to 36 months. Using random effects for households, Bayesian models were used to estimate independent survival by net type (Interceptor®, Life Net®, MAGNet™, Netprotect®, Olyset® Net, PermaNet® 2.0 R, PermaNet® 2.0 C, Yorkool® LN) and by area (rural/urban). In addition to survival, median survival time and attrition of each LLIN brand was determined. Attrition was defined as nets that were missing because they were reported given away, destroyed and thrown away, or repurposed.

**Results:**

Three net types had a proportion of survival above 80% after 24 months: Interceptor®87.8% (95% CI 80–93.4); conical PermaNet® 2.0 86.9% (95% CI 79.3–92.4) and Life Net® 85.6% (95% CI 75–93). At 36 months, conical PermaNet® 2.0 maintained a good survival rate, 79.5% (95% CI 65.9–88.8). The attrition due to redistributed nets showed that the two conical net types (PermaNet® 2.0 and Interceptor®) were more often retained by households and their median retention time was well above 3 years (median survival time = 3.5 years for PermaNet® 2.0 and median survival time = 4 years for Interceptor®). Despite this good retention, Interceptor® had weak physical integrity and its median survival due to wear and tear was below 3 years (median survival time = 2.4 years). The odds ratio of survival was 2.5 times higher in rural settings than in urban settings (OR 2.5; 95% CI 1.7–3.7).

**Conclusions:**

Differences in survival among LLIN may be driven by brand, shape or environmental setting. In this study in Senegal, conical PermaNet® 2.0 were retained in households while rectangular PermaNet® 2.0 had lower retention, suggesting that net shape may play a role in retention and should be further investigated. Distribution of preferred LLIN shape, accompanied by good communication on care and repair, could lead to increased effective lifespan, and allow for longer intervals between universal coverage campaigns.

## Background

Vector control based on use of long-lasting insecticidal nets (LLIN) is a major element for fighting against malaria. For decades, studies have shown that LLIN reduce morbidity and mortality due to malaria [1, 3, 19, 24]. Technology of LLIN was developed in response to the low retreatment rate of conventional nets [11, 19, 40, 41]. Beyond the physical protection offered by bednets, they induce a community effect due to the lethal effect on the vector population [8, 13, 19]. With these results, LLIN became an essential component of health programs around the world [34].

In recent years, many efforts have been undertaken in malaria-endemic areas, particularly in sub-Saharan Africa, to increase distribution and use of LLIN. Manufacturers are reported to have delivered 582 million nets worldwide between 2014 and 2016. Sub-Saharan Africa alone received 505 million nets, compared to 301 million in the previous 3 years (2011–2013) [39]. In Senegal, mass distribution campaigns began in 2010 with an aim to cover all sleeping spaces and increase the use rate of LLIN [25].

Despite these investments in LLIN distribution and use, there are still questions about the effective lifespan of LLIN under local conditions [15, 16, 33]. Since a decade, many programs supported monitor net durability and called for more research on LLIN distributed in different malaria endemic areas [27, 28].

LLIN are nets that retain their insecticidal properties after 20 washes under laboratory conditions [30, 31]. However, the effectiveness of a LLIN is determined not only by its insecticidal effect but also by its physical survival rate in households [7], which includes both retention and the physical condition of the net. Many studies have been done on the evaluation of LLIN durability. Some have shown that LLIN, contrary to implied expectations, may not have an effective lifespan of 3 years [2, 9, 16]. It is important to monitor at what point nets are given away or discarded in addition to the physical condition of the cohort nets still found in the household. Compared to other countries, Koenker and colleagues found that in Senegal, more LLINs were lost in the first year when compared to other countries [18].

As part of the universal coverage strategic framework, WHO recommends mass distribution every 3 years based on an average and effective lifespan of LLINs set by WHO PQ. WHO has issued a recommendation encouraging national programs to monitor and evaluate the performance of LLINs under local field conditions in order to choose the best net profiles to put in place and the period after which they will need to be replaced. Several methodologies have been adopted to understand the durability of LLIN [35]. One key aim in LLIN durability monitoring is to see if campaign distributed nets are still in households. If not, where are the nets and why are they no longer in the household? This study presents an investigation on survival of different LLIN brands in central and western Senegal and their variation in rural and urban settings.

## Methods

### Study area

**Fig. 1:**
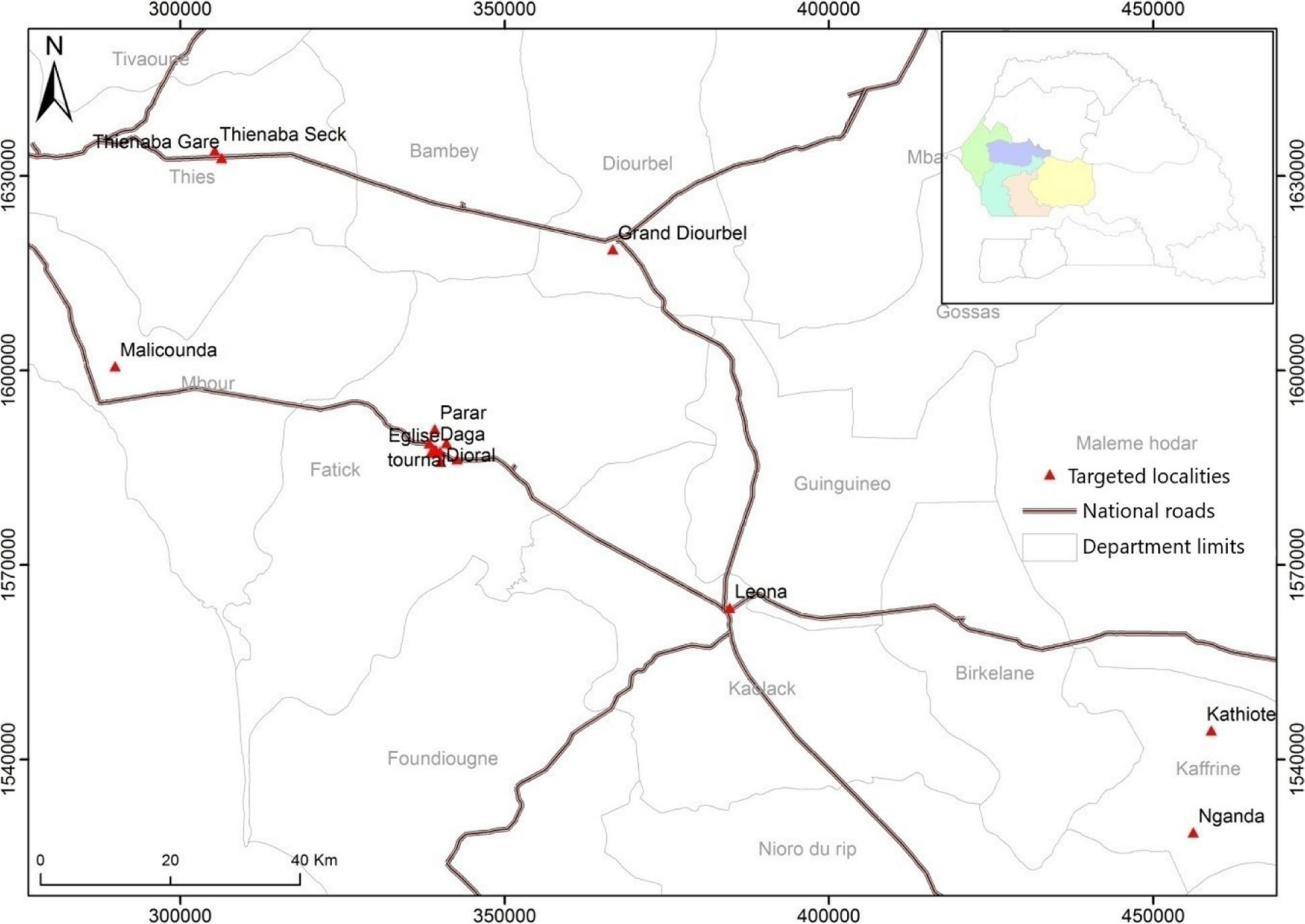
Location of study sites (map created by using ArcGIS software)

In November 2014, the Senegalese National Malaria Control Program (NMCP) supported by the U.S. President Malaria Initiative (PMI), launched a universal coverage campaign in four regions of the central zone (Diourbel, Fatick, Kaffrine and Kaolack) and in March 2015 in a region of the West zone (Thiès). Distribution of LLINs was done in order to obtain urban and rural stratification in different regions. Urban areas were localized in Diourbel and Kaolack respectively represented by districts of Leona and Grand Diourbel. Rural areas were localized in Fatick represented by the villages of Ndiongolor and in Kaffrine represented by the villages of Nganda and Kathiote. The western region, Thiès, was divided into two rural and urban areas represented by Malicounda and Thienaba respectively (Fig. 1). In rural areas, the households visited had generally constituted by straw roofs, banco walls, wooden or metal beds. The urban area was characterised by households with tin or cement roofs, cement walls, wooden or metal beds.

These regions are characterized by a Sahelian, Sahelo-Sudanian or Sudano-Sahelian climate with a long dry season (eight to nine months) and a short rainy season (three to four months). In the central zone, the average annual rainfall is 750 to 800 mm with a rotation farming system of peanuts, millet, maize, cowpeas, etc. In the western zone, the average rainfall is 400 to 500 mm, and gardening is practiced because of water availability a few meters below the ground. The incidence of malaria varies: between 5 and 15 cases per 1000 inhabitants in Thiès and Fatick, between 15 and 25 per 1000 inhabitants in Kaffrine, and over 25 per 1000 inhabitants in Diourbel and Kaolack [26]. *Plasmodium falciparum* is responsible for 90% of malaria infections and the dominant vectors are *Anopheles gambiae*, *An. coluzzii*, *An. arabiensis* and *An. funestus* [23].

#### LLIN distribution

A total of 10,608 nets were distributed during the campaign, one per sleeping space in a numerical order classified by region, LLIN brand and zone. Individual households received one of eight different net product types. LLIN distributed by households was done by randomization and if some brands ran out, others were substituted, so some households received different LLIN brands. All eight LLIN brands distributed (Interceptor®, Life Net®, MAGNet™, Netprotect®,^1^ Olyset® Net, PermaNet® 2.0 R, PermaNet® 2.0 C, Yorkool® LN) were recommended by WHOPES (World Health Organization Pesticide Evaluation Scheme) [38]. The characteristics of different types of LLINs were summarized in Table 1. Five brands (Interceptor®, Netprotect®, Olyset® Net, PermaNet® 2.0 R, PermaNet® 2.0 C) were distributed in central regions (Diourbel, Fatick, Kaolack and Kaffrine) and two others (Life Net®, Yorkool® LN) in the western region Thiès. MAGNet™ was only distributed in Kaolack and Kaffrine.

**Table 1:** Characteristics of different LLIN brands distributed

Brand name	Product type	Insecticide concentration	Denier	Shape	Manufacturer	WHOPES approval
Interceptor®	Alpha-cypermethrin coated on polyester	200 mg/m2	100	Circular	BASF	Full
LifeNet®	Deltamethrin incorporated into polypropylene	8,5 g/kg	100	Rectangular	BAYER	Interim
MAGNet™	Alpha-cypermethrin incorporated into polyethylene	5,8 g/kg	150 ± 7%	Rectangular	V.K.A polymers	Full
Netprotect®	Deltamethrin incorporated into polyethylene	68 mg/m2	118	Rectangular	Bestnet	withdrawn
Olyset® Net	Permethrin incorporated into polyethylene	1000 mg/m2	150	Rectangular	Sumitomo Chemical	Full
PermaNet® 2.0 R	Deltamethrin coated on polyester	55 mg/m^2^	100	Rectangular	Vestergaard Group	Full
PermaNet® 2.0 C	Deltamethrin coated on polyester	55 mg/m^2^	100	Circular	Vestergaard Group	Full
Yorkool® LN	Deltamethrin coated on polyester	55 mg/m^2^	100	Rectangular	Tianjin Yorkool	Full

#### Sampling and survey

A distribution database of households was generated by the NMCP classified by area, type of LLIN, number of LLINs received, and consent of the household head. From this database, random sampling of individual LLIN to follow throughout the 3 years was done. The study was longitudinal with follow-ups at 6, 12, 24 and 36 months. Two hundred fifty to 300 samples of each LLIN brand, stratified by rural and urban areas, were selected during the random sampling at the sixth month post distribution, establishing a cohort of 2222 nets in households. For the random sampling, the nets included in the survey were selected using Stata software (version 11.2) with stratification for the LLIN product distributed. Due to the presence of some large households with many sleeping spaces and many nets, several LLINs (of the same product type) were sampled in these households. After consent from the household head at each round, a member of the team administered the questionnaire to a household respondent aged at least 18 years old. A double marking system was used to identify cohort LLINs: barcodes sewn onto the label and written onto the label with indelible marker in case the barcode was removed. At each follow-up, the surveys were conducted in the households available in the database. Unavailable households (due to absence, exclusion, lack of access, etc.) that were not replaced contributed to the drop in the number of surveyed households over time.

The survey questionnaire was administered using Android tablets with Open Data Kit (ODK) software. Investigators collected GPS coordinates at baseline to map households surveyed. From the tablets, data were sent to a server that hosted the main database. The 6-month data collection took place simultaneously in the 4 central regions and (Diourbel, Fatick, Kaffrine and Kaolack) in May 2015 and in the western region Thiès in September 2015. The 12, 24 and 36 month rounds were conducted in November 2015, 2016 and 2017 in the four central regions and in March 2016, 2017 and 2018 in the western region Thiès.

#### Physical integrity

The physical condition of nets was measured by counting the number of the category of holes that were on the surface of each net. Four categories of holes were defined by the World Health Organization (WHO) [35]: size 1 (0.5–2 cm diameter), size 2 (2–10 cm diameter), size 3 (10–25 cm diameter) and size 4 (> 25 cm diameter). With holes classified in those size categories and counted, a proportionate hole index (pHI) was calculated for each net in order to estimate the physical damage:

$$\mathit{pHI}=\#\textit{size}\hspace{0.25em}1\hspace{0.25em}\textit{holes}+\left(\#\textit{size}\hspace{0.25em}2\hspace{0.25em}\textit{holes}\times23\right)+\left(\#\textit{size}\hspace{0.25em}3\hspace{0.25em}\textit{holes}\times196\right)+\left(\#\textit{size}\hspace{0.25em}4\hspace{0.25em}\textit{holes}\times576\right)$$

The pHI allowed classifying nets into three categories according to arbitrary thresholds:

$$\mathrm{Good}:\;0\;\leq\;\mathrm{pHI}\;\leq\;64;\;\mathrm{Damaged}:\;65\;\leq\;\mathrm{pHI}\;\leq\;642;\;\mathrm{Torn}:\;\mathrm{pHI}\;>\;642$$

#### Data preparation and analysis

Data was cleaned to improve matching of LLIN identification codes (IDs) and household IDs throughout the follow-up. For each survey round, each cohort LLIN was coded as ‘LLIN not found’ (LLIN given away, household not found), ‘LLIN discarded’ (LLIN discarded due to wear and tear-given away, but may still be in use by a neighbor, relative, etc.), or ‘LLIN present’. Data was reorganized into a matrix with household IDs, LLIN IDs as line names, and survey round as column names. Any LLIN present at a time of follow-up was considered to be present in previous follow-up even it was not seen or investigated in the last survey. For example, if an LLIN was registered as present at 24 months, it must have been present in previous follow-ups (6 and 12 months). In the case that an LLIN was recorded as destroyed or used for other purposes, it was still considered to be destroyed even if it was not seen at subsequent investigations. In case of a ‘LLIN not found’ value during a survey before an ‘LLIN discarded’ value, it is unknown whether the net was already destroyed during that survey, or still present. A Bayesian model with Gibbs sampling, capable of dealing with this uncertainty in the data, was used for the analysis. With each iteration, this model assigned the status for the unknown data points with a probability based on the known data points. This model considered the probability of net presence at a survey round with a probability equal to zero if the LLIN was assigned the status of ‘discarded’ in the previous survey round. Thus, a probability of survival since the previous follow-up was calculated for each subsequent follow-up. Using random effects for households, several Bayesian models were used: one model with independent survival estimates by LLIN product type and another model for which environment (rural/urban) was taken as explanatory variable. The Bayesian model was implemented in JAGS from the software platform R (version 3.3.2 from CRAN).

Attrition, the complement of survival, was defined as those nets that were missing because they were reported given away, destroyed and thrown, or repurposed. To better understand what happens to lost or present nets, the survival rate of LLIN was calculated in two different ways: ‘Survival rate’ or proportion of nets surviving in serviceable condition: defined as the proportion obtained by calculating the number of sampled LLINs physically present and usable on the number of LLIN surveyed at time x. The lost LLIN which were given away, sold, or stolen were excluded from the denominator, as their ultimate outcome is unknown. This survival rate accurately reflects the level of loss due to diminishing physical integrity over time. The number of LLIN present and serviceable was calculated by including data on the reason for the loss (either damaged and discarded, or used for other purposes) and the calculation of pHI. LLINs that had a destroyed surface less than 1000 cm^2^ were considered serviceable (0 ≤ pHI ≤642). We used same calculation proposed by the Vector Control Technical Expert Group [37] with some modifications. In this study the denominator did not include all nets from the original distribution but only the selected and surveyed nets at time x. This was due to excluded households during each follow-up, i.e. after sampling for laboratory tests or other obstacles (absence, refusal, no accessibility, etc.). This calculation allowed having a very low denominator at 36 months which can induce a slight increase of survival or retention.

$$\%\;\hspace{0.25em}\textbf{Survival rate}=\frac{\text{Number of LLIN present and serviceable}\hspace{0.25em}\mathrm{at}\hspace{0.25em}\text{time}\hspace{0.25em}\mathrm x.}{\text{Number of LLIN surveyed}\hspace{0.25em}\mathrm{at}\hspace{0.25em}\text{time}\hspace{0.25em}\mathrm x.}\ast100$$

Retention rate: defined as the proportion obtained by dividing the number of sampled LLIN physically present over the number of LLIN surveyed at time x. Lost nets that were damaged and discarded, or used for other purposes are excluded from the denominator. This rate reflects the level of LLIN retention within households.

$$\%\;\hspace{0.25em}\textbf{Retention rate}=\hspace{0.5em}\frac{\text{Number of LLIN present}\hspace{0.25em}\mathrm{at}\hspace{0.25em}\text{time}\hspace{0.25em}\mathrm x.}{\text{Number of LLIN surveyed}\hspace{0.25em}\mathrm{at}\hspace{0.25em}\text{time}\hspace{0.25em}\mathrm x.}\ast100$$

Median survival time was calculated for each LLIN brand based on calculated survival and retention rates. Due to the small number cohort of LLIN found at 36 months, the calculation of the median survival time (in years) was done from data obtained at 12 and 24 months.

$$\mathrm{Tm}=t1+\frac{\left(t2-t1\right)\ast \left(p1-50\right)}{\left(p1-p2\right)}$$

Tm is the median survival time, t1 and t2 represent respectively first and second year, p1 and p2 represent respectively the survival and retention rate in the first and second year [36]. The confidence interval of the median net survival was obtained by applying the formula to the lower and upper limits of p1 and p2, respectively. These who used survival curve proposed by the Vector Control Technical Expert Group, it was observed that projections toward the median should only be attempted if the first time point is at least 85% or lower. In this study, survival curve was not used because some LLIN brands had a first time point (12 months) higher than 85%. The Tm is calculated separately and represented in histograms with-error-bars.

## Results

### Reasons for LLIN being absent

From the initial cohort of 2222 LLIN surveyed at 6 months post-distribution, 1652 were surveyed at 12 months, 1128 at 24 months and 119 at 36 months. Checks on the presence of LLINs showed that at 6 months 2141 nets were present and 81 absent; at 12 months 1366 were present and 286 absent; at 24 months 498 were present and 630 absent; at 36 months 70 were present and 49 absent. The reasons for LLIN being absent are listed in Table 2.

**Table 2:** Reasons for LLIN being absent at 6, 12, 24 and 36 months

Reason for LLIN absence	6 months		12 months		24 months		36 months	
	*N* = 81	%	*N* = 286	%	*N* = 630	%	*N* = 49	%
Damaged and discarded	10	12.35%	67	23.43%	241	38.25%	32	65.31%
Used for other purposes	1	1.23%	8	2.80%	13	2.06%	0	0.00%
Given away	42	51.85%	118	41.26%	143	22.70%	6	12.24%
Stolen	8	9.88%	83	29.02%	200	31.75%	9	18.37%
Exchanged	20	24.69%	10	3.50%	33	5.24%	2	4.08%

The recorded reasons for absence show that the early loss was due to nets being given away to others, representing 51.85% of reasons at 6 months and 41.26% at 12 months. However, at 24 and 36 months, loss was mainly due to damage and theft representing respectively 38.25 and 31.75% of reasons after 24 months and 65.31 and 18.37% of reasons after 36 months.

### Probability of survival of different LLIN brands

**Fig. 2:**
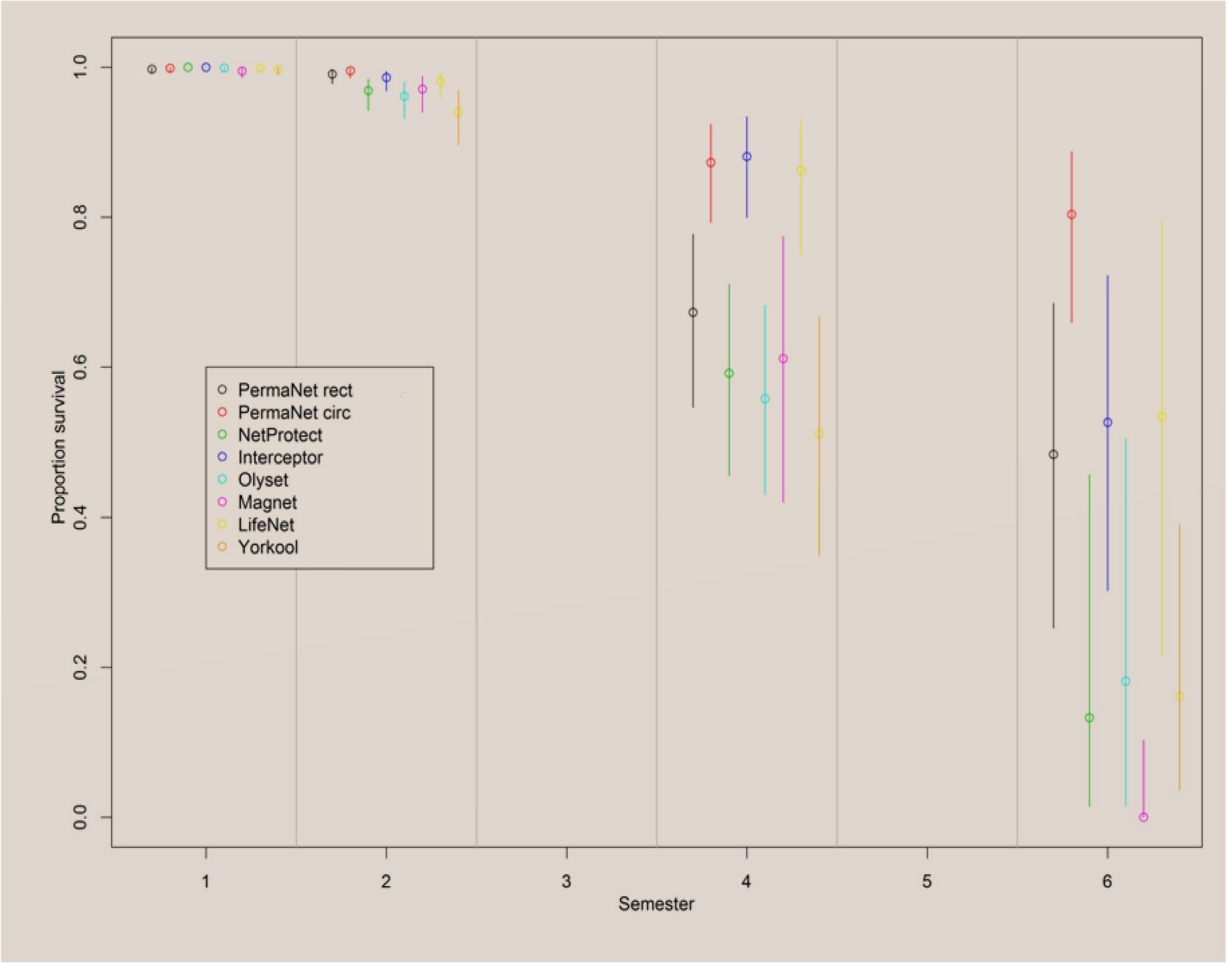
Probability of survival of different LLIN brands. Abbreviations: PermaNet 2.0 cir: PermaNet 2.0 Curcilar, PermaNet 2.0 rect: PermaNet 2.0 Rectangular

Longitudinal monitoring of LLINs in households over time shows that the probability of survival of an LLIN in a household varies from one brand to another and / or from one survey round to another (Fig. 2). The results showed that the survival of all LLINs brands is highest during the first 6 months post-distribution (near 100%). A significant reduction in survival is noted over the subsequent rounds. This loss is greater for some brands than for others. Indeed, 24 months after distribution, a survival rate less than 80% is observed for LLIN brands such as PermaNet® 2.0 R with a probability of 66.9% (95% CI 54.6–77.7), MAGNet™ 60.7% (95% CI 42–77.4), Netprotect® 59% (95% CI 45.5–71), Olyset® Net 55.7% (95% CI 43–68.2) and Yorkool® LN with a probability of 51.2% (95% CI 34.9–66.7). Meanwhile, net survival was higher in Interceptor®, PermaNet® 2.0 C and Life Net® respectively 87.8% (95%CI 80–93.4); 86.9% (95% CI 79.3–92.4); 85.6% (95% CI 75–92.9). After 36 months, only PermaNet® 2.0 C had a probability of survival close to 80% (95% CI 65.9–88.8).

### Impact of zone on the survival of LLIN brands

**Fig. 3:**
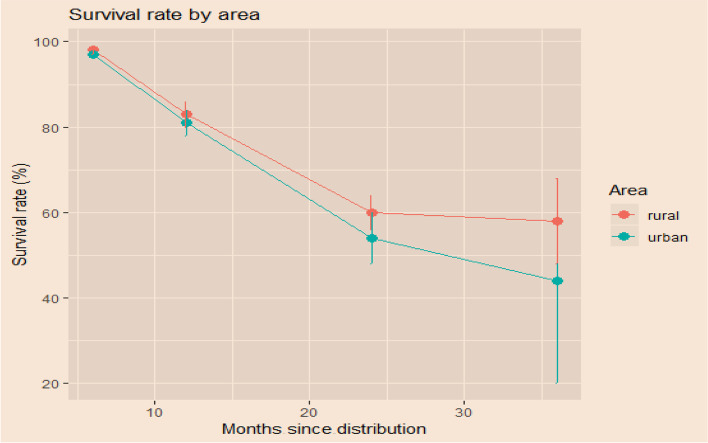
Survival rate in rural and urban areas at 6, 12, 24 and 36 months

**Fig. 4:**
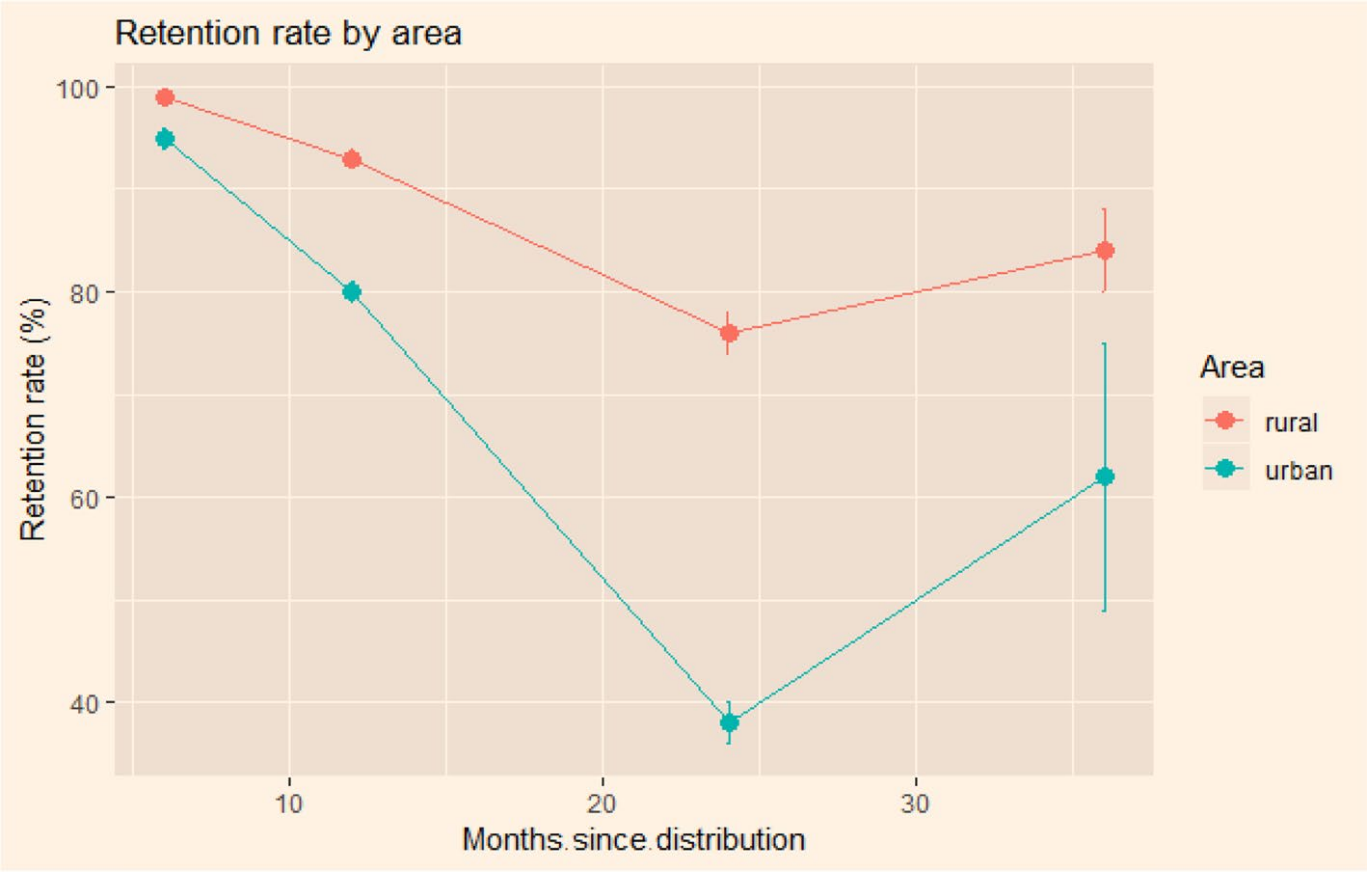
Retention rate in rural area at 6, 12, 24 and 36 months

Pooling LLIN brands together, the survival rate is higher in rural areas compared to urban areas. We also noted that loss due to moving away or giving away is very marked in urban area (Figs. 3 and 4). Beyond the low number of surveyed nets at 36 months, it is important to know that the few nets retained in households are those that were in good condition. Using the urban and rural zones as an explanatory variable, the results of the model with random effects for households showed that the probability of survival of LLINs was twice as high in rural areas compared to urban areas during over the study period. The coefficient was 0.924 (95% CI 0.543–1.316) corresponding to an odds ratio of 2.5 (95% CI 1.7–3.7).

### Survival rates of different LLIN brands

**Fig. 5:**
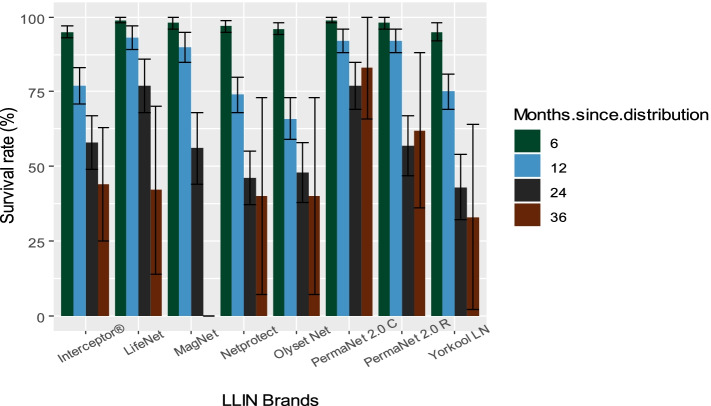
Survival rate of different LLIN brands at 6, 12, 24 and 36 months. Abbreviations: PermaNet 2.0 C:PermaNet 2.0 Circular, PermaNet 2.0 R: PermaNet 2.0 Rectangular

For each LLIN brand, we calculated survival and retention rates and the corresponding median survival time (Tm). Results (Fig. 5) revealed that loss due to destroyed or repurposed nets varied from one LLIN brand to another. At 6 months, Yorkool® LN had an average survival rate of 94.9% (95% CI 91.6–97.2), Interceptor® 95.5% (95% CI 92.2–97.6) and Olyset®Net 95.7% (95% CI 92.5–97.7). At 12 months, the loss is more important in Olyset® Net with an average survival rate of 66.3% (95% CI 59.1–73), in Yorkool® LN with 75.4% (95% CI 68.4–81.6), in Interceptor® with 77% (95% CI 70.3–82.9) and in Netprotect® with 74.3% (95% CI 67.4–80.4). At 24 months, a decrease in survival is noted in almost all LLIN brands distributed excepted Life Net® and PermaNet® 2.0 C which have respectively an average survival rate of 77.3% (95% CI 66.2–86.2) and 76.7% (95% CI 68.1–84).

**Fig. 6:**
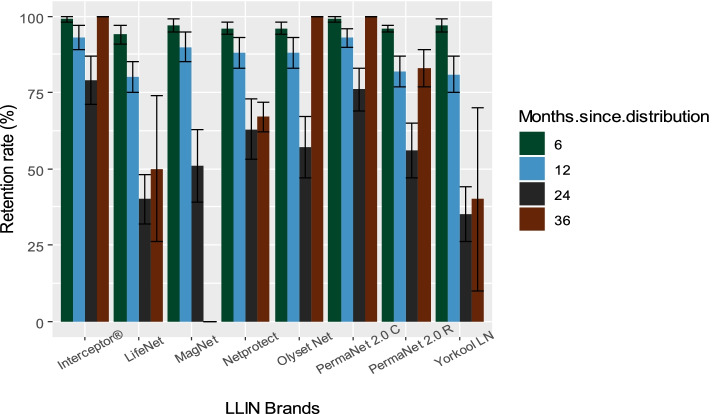
Retention rate of different LLIN brands at 6, 12, 24 and 36 months. Abbreviations: PermaNet 2.0 C: PermaNet 2.0 Circular, PermaNet 2.0 R: PermaNet 2.0 Rectangular

The level of retention also varied from one LLIN brand to another (Fig. 6). At 6 months, loss due to giving away is relatively greater for Life Net® with an average retention rate of 94% (95% CI 90.7–96.4). At 12 months, the retention rate decreases in Life Net® with 80.2% (95% CI 74.3–85.2), Yorkool® LN 81% (95% CI 74.8–86.3) and PermaNet® 2.0 R 82.2% (95% CI 76.5–87.1). At 24 months, a large decrease in retention is noted in all LLIN brands except Interceptor® and PermaNet® 2.0 C which had respectively an average retention rate of 79.1% (95% CI 70.3–86.3) and 76% (95% CI 67.7–83.1).

Survival and retention rates were calculated based on surveyed nets at each time point. Due to a decrease in the number of total surveyed nets at 36 months, a low denominator led to an observed rise in survival and retention rates in some LLIN brands; however, that may be due to the decrease of nets in households.

### Median survival time of LLIN brands

**Fig. 7:**
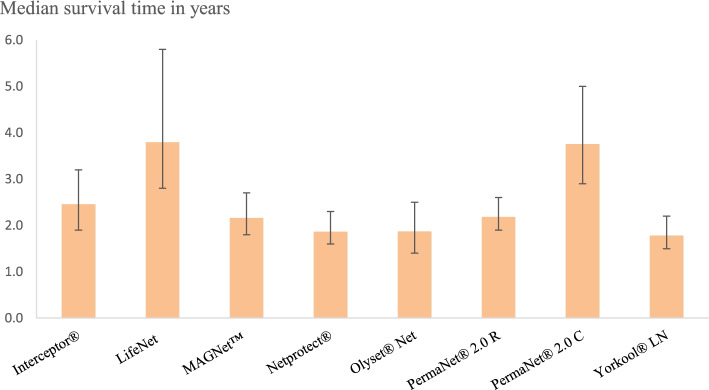
Median survival rate time of different LLIN brands. Abbreviations: PermaNet 2.0 C: PermaNet 2.0 Circular, PermaNet 2.0 R: PermaNet 2.0 Rectangular

**Fig. 8:**
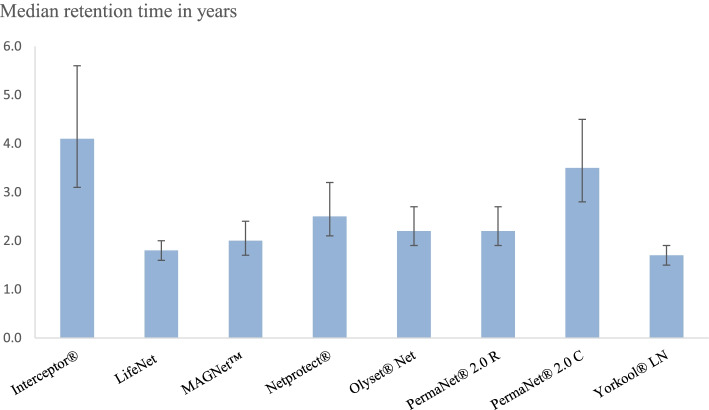
Median retention time of different LLIN brands. Abbreviations: PermaNet 2.0 C: PermaNet 2.0 Circular, PermaNet 2.0 R: PermaNet 2.0 Rectangular

Calculation of median survival time applied to survival rates (loss due to diminishing physical integrity) showed that out of the 8 LLIN brands, only PermaNet® 2.0 C and Life Net® had an estimated median survival time relatively close to 4 years (95% CI 2.8–5.8). Interceptor®, MAGNet™, PermaNet® 2.0 R, Netprotect®, Olyset®Net and Yorkool® LN had a median survival time below 2.5 years (95% CI 1.4–3.2). Calculation of median survival time applied to retention rates revealed that PermaNet® 2.0 C and Interceptor® had respectively a median retention time of 3.5 years (95% CI 2.8–4.5) and 4 years (95% CI 3.1–5.6). Life Net®, MAGNet™, PermaNet® 2.0 R, Netprotect®, Olyset®Net and Yorkool® LN have a median retention time below 2.5 years (95% CI 1.6–3.2) (Figs. 7 and 8).

## Discussion

Currently, there is a growing field of research on efficacy of malaria vector control tools, particularly investigations into survival and retention of long lasting insecticidal nets (LLINs) in households. This study shows that LLIN survival decreases over time. This corroborates a study done in Mozambique where it was found that the proportion of mosquito nets reported as damaged increases every year [22]. Loss is either due to declining physical condition (holes or tears), or to redistribution by giving away, moving away or theft. Loss due to moving away is the most important during the first months post-distribution. These results confirm those of Koenker who found that the majority of lost nets during the first months were given away to other people, and provides additional information on the early loss of LLIN found in Senegal [18]. In Benin, Azondekon et al. 2014 found that majority of lost nets after 6 months were from people moving away [4].

Some LLIN brands developed holes faster than others. Yorkool® LN, Olyset® Net and Interceptor® were observed to develop holes faster than other brands. Other studies have shown poor physical integrity of LLIN due to holes [2, 9, 14, 22]. In the case of Life Net®, we observed better physical integrity, but higher rates of giving away to other people, which may demonstrate the impact of user preferences or acceptability between the different LLIN brands distributed.

Results on LLIN retention show that two nets (Interceptor® and PermaNet® 2.0 C) were the most retained in households. This strong retention might be explained by their conical shape, which is popular in Senegal. In Ethiopia, Baume found that circular nets were much more likely to be used than rectangular ones [6]. Net shape may also explain why conical PermaNet® 2.0 C was well retained in households (median retention time of 3.5 years) while PermaNet® 2.0 R (rectangular), which has the same physical qualities, was found with low retention (median retention time 2.2 years). These PermaNet® 2.0 R (rectangular) results are below those found by Kilian after 3 years at three different sites in Nigeria [17]. This finding suggests that by calculating survival separately and combining data with survey results on net shape may provide an explanation for the differential retention of net types.

The Bayesian model is an excellent survival analysis tool which showed availability of Life Net® despite redistribution. However, it was unable to detect poor physical integrity of Interceptor®. In this context, it can be said that loss of nets due to moving away does not constitute a real loss, as the outcome of the net is unknown (and the net is likely to be in use somewhere else). That said, it is important to use different methodologies to control survival rate to better understand the factors linked to net loss.

These results show that the simple acquisition of holes does not solely determine the loss rate of LLINs in households. Despite its poor physical condition, Interceptor®, was still retained in households. It is also important to note that users may perceive nets as ‘very torn’ before they reach a pHI of 642, influencing loss due to discarded nets (survival rate). We found that some LLIN in good physical condition were thrown away or used for other purposes; this was the case for MAGNet™. Loll found that in Senegal, the determining factor to the end of life of nets was a poor perception of physical integrity [20]. The observed high retention of conical LLIN leads us to assume that a less preferred LLIN with holes is more likely to be discarded or used for other purposes than a preferred LLIN with the same physical condition. Our results may help to explain results from Batisso as well as Helinski et al. [5, 14] to explain their respective results: finding that a third of discarded nets were less than 12 months and that a large number of nets were discarded 6 months post-distribution with a paradoxical reason of ‘very old or very torn ’[5, 14]. In Ethiopia, after 1 year of distribution, some users were concerned about the condition of their mosquito nets saying that: «Mosquito nets are old and have holes, they do not protect against mosquitoes » [6]. These findings demonstrate that the survival time of LLIN in households depends on a set of intrinsic and extrinsic factors, including the type and brand of LLIN distributed.

The longevity of LLIN is likely to differ from one region or culture to another [32]. Beyond the difference between LLIN brands, the ‘zone’ factor can also influence survival rates. This study showed that loss, whether due to wear and tear or give away, was much greater in urban than in rural areas. A study in Rwanda found that the loss of physical integrity was more pronounced in urban localities [12]. Another study carried out in four malaria-endemic countries showed that loss due to giving away nets was greater in urban compared to rural areas [18]. High retention in rural areas might be explained firstly by their attachment to net use [18], and secondly by a lack of choice forcing households to use or retain the available LLIN. Several studies have found that the burden of malaria weighs more in rural areas generally characterized by poverty [10, 21, 29]. The considerable net loss noted in urban areas may be explained by the availability of other nets (purchased in local markets) and other means of combating nuisance (mosquito coils, insecticide sprays and screened windows).

### Limitations

This study has some limitations caused by small number of surveyed nets which decreased over time. It would be more rigorous to replace excluded households in order to keep a large sample of LLIN brands.

## Conclusion

The concept of net durability must not be defined only by the availability of insecticidal effect but also by the physical availability of nets in households. This study suggests that, in Senegal, LLIN survival decreases over time with a more pronounced reduction for some brands. Survival calculated according to two parameters showed that median lifespan of LLIN ranged from 2.5 to 4 years. The loss of nets in urban areas could be filled by social marketing, and distribution channels more focused in rural areas. Future assessments of net durability, including intrinsic and extrinsic factors of net, acceptability, and household behaviors and attitudes may provide explanations to better understand the effective lifespan of LLIN. Distribution of preferred LLIN shape, accompanied by good communication on care and repair, could lead to increased effective lifespan, and allow for longer intervals between universal coverage campaigns.

## Data Availability

The data used and analyzed during the current study are available from the corresponding author on reasonable request.

## Abbreviations

RBM: Roll Back Malaria
VCWG: Vector Control Working Group
LLIN: Long Lasting Insecticidal Nets
Tm: Median Survival Time
PMI: President’s Malaria Initiative
NMCP: National Malaria Control Program
CNERS: National Committee of Ethics and Health Research
WHO: World Health Organization
WHOPES: World Health Organization Pesticide Evaluation Scheme
ODK: Open Data Kit
pHI: Proportionate Hole Index
IDs: Identification codes
N: Sampling nets
PermaNet® 2.0 R: PermaNet® 2.0 Rectangular
PermaNet® 2.0 C: PermaNet® 2.0 Circular
CI: Confident Interval

## Footnotes

1 The interim recommendation of Netprotect® has been withdrawn but the WHOPES working group recommended that programs should continue to assess its durability under local conditions to obtain more information about product quality [36].
